# The Use of Seleniol Injections for Sarcoma

**Published:** 1913-02-22

**Authors:** 


### THE USE OF SELENIOL INJECTIONS FOR SARCOMA.

At the meeting of the Section of Laryngology* on Friday, the 7th inst., Mr. C. W. M. Hope showed a case of great interest, in which a striking disappearance of a malignant mass was brought about by injection of seleniol. The patient was a man, æt. twenty-five, who attended hospital on September 7 with a swelling of the right tonsil of two months' duration. When seen, the tonsil was of the size of a hen's egg, was hard to the touch, and on its anterior surface were two ulcers. On September 17 preliminary laryngotomy was performed, and then the mass was enucleated *viâ* the mouth. The microscopist reported that it was mixed-celled sarcoma. On November 11 slight induration could be felt beneath the upper end of the right sterno-mastoid. Nearly a month later the mass was found to be increasing, extending from the base of the mastoid process to the upper border of the thyroid cartilage, and as the head movements were becoming limited it was cut down upon. The tissues were indurated, and a gland was removed which lay very deep in front of the transverse process of the second cervical vertebra. It was broken-down and necrotic. The wound healed by first intention. The microscopic report was that it was sarcoma undergoing necrotic changes. Mr. Hope then injected into the mass 3 c.c. of seleniol on December 14, 17, 21, 24, and 28, and on January 10 the deep mass was found to have disappeared, and there remained only some thickening in the scar, which was freely movable. The patient now looked and felt very well, and his weight had increased 5 lb. in two weeks.

Seleniol is described as an electrolytic colloid of the metal selenium; it can be injected subcutaneously, intravenously, or directly into the tumour. There is no toxic effect, and the growth is said to either be absorbed or to liquefy. Mr. Hope showed a second case, much improved by the same substance, of what was diagnosed as swelling above a malignant ulcer of the larynx.

At the same meeting Mr. Douglas Harmer exhibited two remarkable cases. The patient, a lady, had for years suffered from hay-fever. Following a severe cold in November last, she had a persistent discharge of mucus from the right side of the nose, and occasionally a thick membranous cast from the back of the throat, the latter of brownish-yellow colour, and of the appearance of wet blotting-paper. There had been violent attacks of sneezing, but no bleeding. The right side of the nose was blocked by œdematous mucous membranes. Cocaine and adrenalin caused some subsidence, but a good view of the nose could never be obtained. The post-nasal space was also full of mucus, and in the pharynx was a quantity of frothy saliva. The right antrum was dull. Her general health was bad, and she had the appearance of having been poisoned. She had occasional headaches, and neuralgic pains around the right eye. Under general anæsthesia an intranasal opening was made into the antrum. After considerable washing out, small lumps of brownish membrane began to appear; but the membrane was very difficult to dislodge. Blood-agar plates showed a copious growth of *Aspergillus fumigatus* at 37° C. The original secretion revealed mycelium. After four weeks' varying treatment, iodide of sodium was given internally, commencing with 2-gr. doses, and rapidly increasing to 20 gr. three times a day. The antrum was now washed out as often as possible with five volumes peroxide of hydrogen. At the end of ten days all the discharge had ceased; the general health improved, and she had remained well a fortnight.

The next patient was a lady who had suffered from rhinitis for many years, for which she had had douches of various kinds. Recently, on a recommendation from a friend, she had used a solution of a patent sea-salt, but following its employment she had great irritation and attacks of sneezing. On washing out she discovered two specimens of a sea monster, *Artemia solina.* The eggs are found in sea-water, and the animal flourishes when the salt reaches a high degree of concentration.

* Royal Society of Medicine.

